# Fast-track management of pneumothorax in laparoscopic surgery

**DOI:** 10.4103/0019-5049.76564

**Published:** 2011

**Authors:** Raviraj Raveendran, Hari Narayana Prabu, Sarah Ninan, Sathish Darmalingam

**Affiliations:** Department of Anaesthesiology, Christian Medical College and Hospital, Vellore, Tamil Nadu, India

Sir,

A 16-year-old ASA risk I boy with pelviureteric junction obstruction was scheduled for elective laparoscopic left pyeloplasty. After establishing routine monitoring (electrocardiogram, pulse oximetry, non invasive blood pressure), a standard general anaesthesia was given with thiopentone sodium, fentanyl and isoflurane. The trachea was intubated with a 7.5-size cuffed endotracheal tube (ETT), which was facilitated by vecuronium. The ETT position was confirmed by auscultation and end-tidal carbon dioxide (ETCO_2_) trace, and later the patient was positioned for surgery (right lateral). Intermittent positive pressure ventilation (IPPV) was maintained using air and oxygen mixture. The initial ventilator parameters were tidal volume 500 ml, respiratory rate 12/min, I:E ratio 1:2, and the peak inspiratory airway pressure (PIP) was noted as 17 cm H_2_O. Within 10 min of pneumoperitoneum initiation with a pressure limit of 15 mmHg (20.4 cm H_2_O), the PIP rose to 40 cm H_2_O. Manual ventilation showed a tight bag and auscultation revealed there was no air entry on the left haemithorax. Presuming that it could be due to right endobronchial migration of the ETT due to pneumoperitonium, the ETT was pulled out 2 cm. But there was no improvement in the airway pressure as well as in air entry on the left-side chest. At the same time, there was neither desaturation nor haemodynamic changes except the ETCO_2_level which rose to 45 mmHg. In order to treat hypercarbia, the total minute ventilation was increased by stepping up the respiratory rate.

A fiberoptic bronchoscope examination was performed to rule out endobronchial placement and obstruction due to secretions. With a baseline PIP 17 cm H_2_O and pneumoperitoneal pressure 20 cm H_2_O, the expected PIP should have been less than 37 cm H_2_O, but it happened to be 40 cm H_2_O. This raised the suspicion of pneumothorax. This scenario was discussed with the surgeon, and a traumatic puncture in the left-side diaphragm was ruled out by laparoscopic examination. Then the surgeon was advised to reduce the insuffalation pressure from 15 mmHg to 12 mmHg, which reduced the PIP from 40 cm H_2_O to 35 cm H_2_O. This helped to improve the compliance of the left lung, but still the air entry was less. Since the patient was haemodynamicaly stable and there was no desaturation, the surgery was continued laproscopically for next 2 h without any intervention.

**Figure 1:**
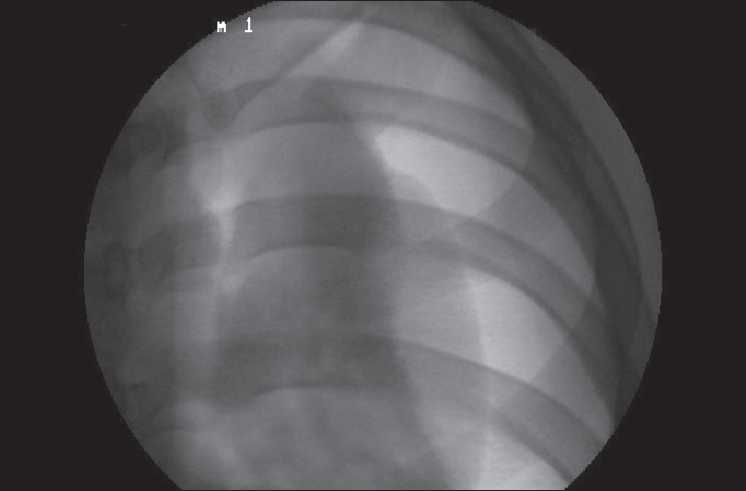
Left-side pneumothorax with the collapsed lung

At the end of the surgery, the presence of pneumothorax was confirmed by an image intensifier Figure 1. On maintaining the IPPV, a 16-G IV cannula was placed in the third intercostal space at the midclavicular level. This helped to reduce the pnuemothorax within 2-3 minutes, and the lung expansion was confirmed with serial films using the image intensifier. After confirming the adequate lung expansion, the patient was extubated and the postoperative period was uneventful.

Pneumothorax during laparoscopy surgery is not uncommon, with a reported incidence of 0.01–0.4%.[1–3] The mechanism behind the pneumothorax is traumatic puncture in the diaphragm or spontaneous pneumothorax due to carbon dioxide entering the mediastinum through the aortic and oesophageal hiatuses of the diaphragm, later rupturing into the pleural space. In our case, since a traumatic puncture in the diaphragm was ruled out, the possible mechanism should be the carbon dioxide entering the congenital weak points or defects in the diaphragm. The high peumoperitoneal pressure (15 mmHg) should have hastened the initiation of pneumothorax which rose the PIP within 10 minutes. Most of the reported cases were diagnosed by the postoperative desaturation or intraoperative haemodynamic instability.[4–6] In our case, an early identification and the limitation of the pneumoperitonium pressure helped to avoid the haemodynamic instability. Carbon dioxide is an easily diffusible gas, so an inter costal drain (ICD) insertion is unnecessary in a haemodynamically stable patient. Image intensifier is easily accessible equipment in the operation theatre, which not only helped to confirm the pneumothorax, but also to monitor the expansion of the lung after needle thoracotomy.
